# The identification of a PRKACA duplication at 19p13.12 in a female with PPNAD and thyroid carcinoma after a 20-year diagnostic journey

**DOI:** 10.1007/s10689-026-00594-9

**Published:** 2026-08-29

**Authors:** Marjoleine F. Broekema, Florian Violon, Patricia Vaduva, Maartje Vogel, Nicole de Leeuw, Fenne Komdeur

**Affiliations:** 1 Department of Human Genetics, Amsterdam UMC, Academic Medical Center, Amsterdam, the Netherlands; 2 Department of Pathology, Hôpital Cochin, Assistance Publique Hôpitaux de Paris, Paris, France; 3 Genomics and Signaling of Endocrine Tumors, Institut Cochin, INSERM U1016, CNRS UMR 8104, Université Paris-Cité, Paris, France; 4 Department of Human Genetics, Radboud university medical center, Nijmegen, the Netherlands

## Abstract

Rare pathogenic variants affecting components of the evolutionary conserved cAMP/protein kinase A (PKA) signalling pathway are implicated in a spectrum of adrenocortical disorders. Germline inactivating *PRKAR1A* variants leading to constitutive PKA activation, underlie primary pigmented nodular adrenocortical disease (PPNAD) in Carney complex. More recently, several patients with PPNAD and other types of bilateral nodular adrenal disease (BNAD) have been diagnosed with rearrangements in chromosome 19 at band p13.12, resulting in copy number gains of the entire *PRKACA* gene. Here, we report the identification of a 19p13.12 rearrangement, resulting in a duplication of the *PRKACA* gene, in a 32-year-old female from whom the genetic aetiology remained unknown for 20 years. Furthermore, we provide an updated overview of patients with *PRKACA* germline copy number gains that have previously been reported in the literature.

## Introduction

Cushing syndrome is an endocrine disorder caused by endogenous hypercortisolism. Clinical features include muscle weakness, hypertension, diabetes mellitus, and osteoporosis, and characteristic physical signs as moon facies, buffalo hump, abdominal striae, obesity, and hirsutism [1]. Depending on the source of cortisol excess, Cushing syndrome is divided in two entities: ACTH-dependent and ACTH-independent [1]. The latter results from primary cortisol hypersecretion and may be caused by bilateral nodular adrenal disease (BNAD) [2], including both macro- and micronodular forms, cortisol-producing adenomas, and adrenocortical carcinoma.

Primary pigmented nodular adrenocortical disease (PPNAD) is a type of BNAD characterized by multiple brown pigmented micronodules of the adrenal cortex and is frequently associated with Carney complex [3]. Carney complex is a rare genetic condition featuring endocrine and non-endocrine tumours as pituitary neuroendocrine tumours, cardiac, cutaneous and breast myxomas, lentiginosis and other abnormalities. Regular screening for myxomas and (endocrine) tumours is recommended for patients with Carney complex.

Inactivating variants in the *PRKAR1A* gene underlie Carney complex [4, 5]. *PRKAR1A*, situated at band q24.2 of chromosome 17, encodes for the main regulatory subunit of protein kinase A (PKA). Inactivation of PRKAR1A results in constitutive activation of the PKA signalling pathway due to the lack of regulation of the main catalytic subunit of PKA, encoded by the *PRKACA* gene [6]. In contrast to pathogenic loss-of-function variants in *PRKAR1A* that lead to the full spectrum of findings that are associated with Carney complex, pathogenic gain-of-function variants in the *PRKACA* gene only lead to benign adrenal tumours. More recently, several patients with BNAD and PPNAD have been described with rearrangements in 19p13.12, resulting in copy number gains of the entire *PRKACA* gene [7–13].

Here, we report the identification of a tandem duplication in 19p13.12, resulting in a total of three copies of the *PRKACA* gene, in a 32-year-old female with PPNAD [14] and thyroid carcinoma, whose underlying genetic cause remained unknown for 20 years. The duplication was inherited by her newborn daughter. Furthermore, we provide an updated overview of patients with *PRKACA* germline copy number gains that have previously been reported in the literature.

## Methods

### Copy number variant analysis

DNA from the proband and her parents was extracted from peripheral blood samples collected in EDTA tubes. Genome wide Copy Number Variant (CNV) analysis in the proband was performed on 100 ng DNA using the CytoScan XON array platform (Thermo Fisher Scientific, Waltham, MA, USA), that contains 6.85 million empirically selected probes for genome wide CNV analysis (with a practical resolution of ~ 100 kb) and which has excellent exon coverage for the reliable detection of intragenic CNVs. CNVs were interpreted in the Chromosome Analysis Suite (ChAS) data analysis software (human genome build GRCh37) and classified using the classification guidelines of ACMG [15].

Carrier testing in the parents was done by CNV in Whole Exome Sequencing (WES) data analysis. For this, exome sequencing was performed using the Twist Exome 2.0 plus Comprehensive Exome Spike-in Kit for enrichment (TWIST Bioscience, San Francisco, CA, USA) after which sequencing was done on an Illumina NovaSeq 6000 sequencer using 2 × 150 bp pair-end sequencing at 100x coverage (Illumina, San Diego, CA, USA). Alignment to the GRCh37 reference genome was accomplished using the Burrows-Wheeler Aligner (BWA) and copy number variant (CNV) were called by the CoNIFER algorithm as previously described [16].

Further characterisation of the copy number gain in the proband was done by region-specific analysis of genome sequencing data. The diagnostic genome sequencing (GS) procedure was followed as previously described [17], using 150 bp paired-end short-reads. In brief, 1000 ng DNA was used for library preparation according to the Illumina DNA PCR-free protocol (Illumina, San Diego, CA, USA), and DNA was tagmented to an average insert size of 450 bp using bead-linked transposomes. Sequencing was performed on an Illumina NovaSeq 6000 sequencing system to an anticipated genome wide coverage of at least 30-fold. Sequencing reads were aligned to the human reference genome GRCh38 by BWA, and variant calling was done using the Illumina’s DRAGEN Bio-IT Platform.

## Results

The proband and her family members gave consent for the publication of non-identifiable details. The proband is a 32-year-old female of European descent who was referred to the department of Human Genetics in the Amsterdam University Medical Center in Amsterdam.

The proband initially presented with type 1 diabetes (T1DM) at the age of 10 years [14]. During a routine consultation for T1DM at the age of 12 years Cushing syndrome was suspected as she presented with a Cushingoid features, including moon facies, hirsutism, and centripetal obesity. She was hypertensive (130/74 mmHg) and her T1DM was difficult to manage with increasing Hb1Ac. Biochemical evaluation confirmed mildly hypercortisolism (urinary free cortisol of 263 nmol/24 h; reference value: 30–250) with a disturbed nychtemeral serum cortisol rhythm, serum cortisol being 547 nmol/l in the morning and 669 nmol/l at 23.00 h. There was a lack of response to low dose (0.5 mg for 48 h) and high dose (2 mg for 48 h) dexamethasone suppression test. ACTH was suppressed, whereas there was a paradoxical increase in the 24 h urinary free cortisol at the end of the test. The abdominal MRI showed no abnormalities of the adrenal glands. Adrenal cortex scintigraphy using 131I-norcholesterol, showing a symmetrical accumulation of noriodocholesterol in both adrenal glands, led to the diagnosis of primary pigmented nodular adrenocortical disease (PPNAD) [14].

As PPNAD is a common characteristic of Carney complex, our patient benefited from a complete screening. The thyroid and cardiac ultrasounds were normal, and no other clinical signs of Carney complex were identified in the patient and her family members. She was treated with a bilateral adrenalectomy. Pathological examination confirmed PPNAD as the adrenal glands contained numerous micronodules composed of large eosinophilic cells that contain focal pigmented deposits of lipofuscin. At the time of the PPNAD diagnosis (2005) no pathogenic variant of *PRKAR1A* was identified by Sanger PCR sequencing in the patient and her parents [14]. The immunoexpression of *PRKAR1A* was normal in the adrenal nodules (data not shown).

At the age of 19 years the patient was diagnosed with hypothyroidism due to Hashimoto disease. At the age of 29 years, she underwent thyroid surgery after she was identified with papillary thyroid carcinoma of the classical subtype. She had a subsequent complementary thyroidectomy and treatment with I-131. The clinical diagnosis Carney complex was considered.

**Fig. 1:**
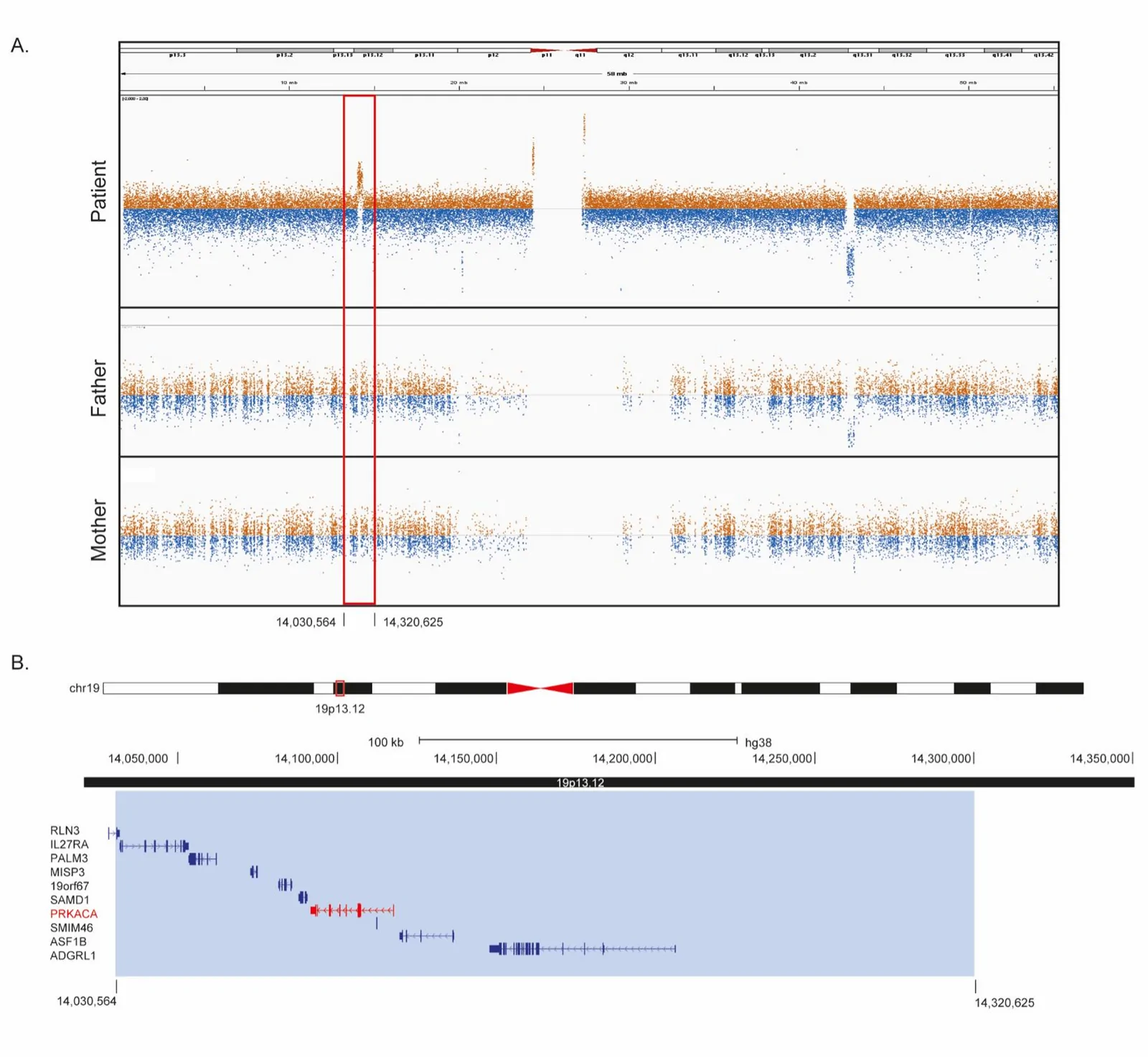
The identification of a *de novo PRKACA *duplication on chromosome 19 at band p13.12.**A** Screen shot from the Integrated Genome Viewer (IGV) of chromosome 19, showing in the upper half of the figure a tandem duplication of 290 kb at 19p13.12 detected in genome sequencing data, containing the *PRKACA* gene (within in the red square), in the proband. The proband’s parents, were tested negative for this 19p13.12 duplication based on CNV in WES data analysis as shown in the lower half of the figure. On the Y-axis is the relative score for each target that is determined after comparing each target value with the average from a reference pool (test over reference ratio). Target values > 0 are in red and target value < 0 in blue. A normal copy number of 2 falls within a range between − 0.4 and 0.4 for genome sequencing calls and between − 1.6 and 1.6 for exome sequencing calls. A value > 0.4 respectively 1.6 indicates a copy number gain, a value < -0.4 respectively − 1.6 indicates a copy number loss. In the middle part of the figure there is no coverage, because this is the centromeric region of chromosome 19 that does not have unique sequences. In the long am of chromosome 19 a common, benign loss of ~ 425 kb in 19q13.2q13.31 is visible in the proband and her father. **B** Screen shot of the UCSC genome browser (http://genome.ucsc.edu/) GRCh38/hg 38 of the duplicated 19p13.12 region, with *PRKACA* indicated in red

Initially, no (likely) pathogenic variants were identified in the proband by targeted sequencing of 158 cancer-predisposing genes and trio-exome sequencing with parents. The CytoScan XON array revealed a constitutional interstitial copy number gain on chromosome 19 at band p13.12. With subsequent genome sequencing the gain was further characterized and shown to be a tandem duplication of 290 kb in 19p13.12 (seq[GRCh38] dup(19)(p13.12p13.12)dn - NC_000019.10: g.(1403563_14030564)_(14320625_14320626)dup). The proband’s father and mother, who do not show any sign of either PPNAD or Carney complex, tested negative for the 19p13.12 duplication (Fig. 1A). Although constitutional germline mosaicism for the tandem duplication in 19p13.12 – which has been described before [10] - in one of the parents cannot be fully excluded, the obtained test results suggest that this 19p13.12 duplication has occurred *‘de novo’* in the proband.

The duplication contains 10 genes (Fig. 1B), including the *PRKACA* gene, and has not been described in the literature before and is absent in the genome Aggregation Database (GnomAD (v4.1.0); http://gnomad.broadinstitute.org). Other 19p13.12 duplications affecting the *PRKACA* gene locus (OMIM #615830) underly ACTH-independent Cushing syndrome. Those 19p13.12 duplications are associated with a spectrum of adrenocortical disease, including micro- and macronodular bilateral adrenocortical disease (Table 1) [7–11]. No genetic changes have been identified by XON array and genome sequencing in the 17q24.2 region harbouring the *PRKAR1A* gene. Therefore, the identified *de novo* duplication in 19p13.12 is considered to be the underlying cause of Cushing syndrome in our patient. Based on these findings, the initial clinical suspicion of Carney complex was reassessed and subsequently rejected.

**Table 1:** Overview of *PRKACA* duplications reported in literature

Family	Patient	Age of presentation (y)	Age of genetic diagnosis (y)	Sex	Initial presentation	Adrenal findings	Adrenalectomy	Other findings	Chromosomal array analysis^†^	PRKACA	References
1	1 (index)	13	32	F	Cushing syndrome	PPNAD	Bilateral	Papillary thyroid carcinoma (29)	Duplication of *PRKACA* in 19p13.12 (g.1403564–14320626)x3 ~ 290 kb	No	Current study
	2 (daughter patient 1)		0	F	No	No			Duplication of *PRKACA* in 19p13.12 (g.1403564–14320626)x3 ~ 290 kb		Current study
2	3 (index)	8		M	Cushing syndrome	BAH	Bilateral	No	Duplication of *PRKACA* in 19p13.12 Unbounded duplication 2.7 Mb	No	[7–9]
3	4	35		F	Cushing syndrome	PMAH	Bilateral (contralateral adrenalectomy 8 years after unilateral surgery)	Breast carcinoma (49)	Duplication in of *PRKACA* in 19p13.2 Unbounded duplication 616 kb	No	[7–9]
	5 (son patient 4)	23		M	Avascular necrosis of the left femoral head with hypertension	PMAH	Unilateral	No	Duplication of *PRKACA* in 19p13.2 Unbounded duplication 616 kb	No	[7–9]
4	6 (index)	2		M	Large at birth, transient hypoglycaemia, macroglossia	iMAD	Bilateral	No	Triplication of *PRKACA* in 19p13.13p13.12 Complex genomic gain of ~ 294 kb in 19p13.13p13.12 with distal duplication followed by proximal triplication. *PRKACA* was in the triplicated fragment.	No	[7–9]
5	7 (index)	2		M	Cushing syndrome	iMAD and/or PMAH	Bilateral	No	Triplication of *PRKACA* in 19p13.13p13.12 Complex genomic gain of 551 kb in 19p13.13p13.12 with distal duplication followed by triplication. *PRKACA* was in the triplicated fragment.	No	[7–9]
6	8 (index)	0.25		M	Cushing syndrome and cutaneous mucinosis	Bilateral non-pigmented micronodular adrenal hyperplasia	Bilateral	Small calcification in left testis, mild left ventricle hypertrophy.	Duplication of *PRKACA* in 19p13.13 p13.12 mosaicism^‡^ The variant allele frequency was in the affected tissues was not reported. Unbounded duplication ~ 900 kb	No	[10]
7	9 (index)	8	22	F	Carney complex	PPNAD	Unilateral	Multiple echoless nodules in the thyroid. Lentigines in face at 8 y.	Duplication of *PRKACA* in 19p13.2 p13.12 (g.13862572–14288606)x3 ~ 426 kb	No	[11]
8	10 (index)	8	8	F	Cushing syndrome	PPNAD	Bilateral	No	Duplication of *PRKACA* in 19p13.12, ∼24.97 Kb (g.(? 14092580)(14117547_? ))x3		[12]
9	11 (index)	18		F	Cushing syndrome	PPNAD			Duplication of *PRKACA* in 19p13.12, (g.13849055–14192940)x3	No	[13]
10	12 (index)	32		F	Cushing syndrome	PPNAD	Bilateral		Duplication of *PRKACA* in 19p13.12, g.14211410_14211411ins14065387_14211410inv	No	[13]
	13 (sister patient 12)	25		F	Cushing syndrome	PPNAD			Duplication of *PRKACA* in 19p13.12, g.14211410_14211411ins14065387_14211410inv	No	[13]
	14 (niece patient 12)	13		F	Cushing syndrome	PPNAD			Duplication of *PRKACA* in 19p13.12, g.14211410_14211411ins14065387_14211410inv	No	[13]
11	15 (index)	9		F	Cushing syndrome	PPNAD			Duplication of *PRKACA* in 19p13.12, (g.13849055_14192940)x3	No	[13]
	16 (relative patient 15)	11		M		PPNAD			Duplication of *PRKACA* in 19p13.12, (g.13849055_14192940)x3	No	[13]
	17 (father patient 15)	28		M	Cushing syndrome	PPNAD			Duplication of *PRKACA* in 19p13.12, (g.13849055_14192940)x3	No	[13]
	18 (sister patient15)	35		F	Cushing syndrome	PPNAD			Duplication of *PRKACA* in 19p13.12, (g.13849055_14192940)x3	No	[13]
12	19	34		F	Cushing syndrome	PPNAD			Duplication of *PRKACA* in 19p13.12, g.14042330_14167117dup	No	[13]
13	20	15		M	Cushing syndrome	PPNAD			Duplication of *PRKACA* in 19p13.12, g.14167117_14167118insGCC	No	[13]
	21 (sister patient 20)	27		F	Cushing syndrome	PPNAD			Duplication of *PRKACA* in 19p13.12, g.14167117_14167118insGCC	No	[13]
	22 (father patient 20)			M		No			Duplication of *PRKACA* in 19p13.12, g.14167117_14167118insGCC	No	[13]
14	23 (index)	12		F	Cushing syndrome	PPNAD			Unbounded *PRKACA* duplication	No	[13]
15	24 (index)	17		F	Cushing syndrome	PPNAD			Unbounded *PRKACA* duplication	No	[13]
16	25 (index)	23		M	Cushing syndrome	PPNAD			Duplication of *PRKACA* in 19p13.12, g.14935704_14935705ins(13928906_14935704inv)	No	[13]
	26 (mother patient 25)			F		No			Duplication of *PRKACA* in 19p13.12, g.14935704_14935705ins(13928906_14935704inv)	No	[13]

^†^ GRCh38

^‡^ Duplication was present in skin, absent in blood

Shortly after the genetic diagnosis, the patient became pregnant, and a daughter was delivered by cesarean section at 35 weeks due to labor arrest and fetal distress. The girl inherited the *PRKACA* duplication from her mother. Because she exhibited a laterality defect suggestive of left isomerism, including bilateral left atrial morphology, abnormal venous return, polysplenia, and intestinal malrotation, exome sequencing of 103 and 423 genes associated with heterotaxia syndrome and congenital heart disease, respectively, was additionally performed. No (likely) pathogenic variants were identified. The cause of the laterality defect in the daughter remains unknown.

## Discussion

Here we present the identification of a *de novo* tandem duplication in 19p13.12, encompassing the entire *PRKACA* gene, in a female with PPNAD and thyroid carcinoma. As the clinical phenotype resembles to previous reported patients with 19p13 duplications, this duplication most likely provides the molecular basis for her disease.

So far, 26 patients and relatives (16 females and 10 males), including our patient and her daughter, from 16 families have been reported in the literature with a copy number gain in 19p13.12, affecting the entire *PRKACA* gene (Table 1). All pathogenic rearrangements encompassed the entire *PRKACA* gene and most affected individuals presented with Cushing syndrome. Although PPNAD was the predominant adrenal phenotype, PMAH, iMAD, bilateral adrenal hyperplasia, and mixed adrenal phenotypes were also reported. Three individuals (patient 2, 22, and 26) had not developed an adrenal phenotype at the time of reporting. These included the newborn daughter of the index patient in the current study, the father in family 11, and the mother in family 16 (Table 1).

The age of presentation ranges from four months to 35 years and the duplication sizes differ from approximately ~ 25 kb (patient 10) to 2.7 Mb (patient 3). Some genomic rearrangements are more complex, involving inversions with concomitant duplication (families 10 and 16) or triplication of the *PRKACA* gene (patient 6 and 7). The observation that the two patients with the smallest and the largest duplication had similar adrenal phenotypes, suggests that the phenotype is largely driven by increased *PRKACA* dosage, with a limited contribution from genes in the surrounding genomic region. This is underscored by the observation that the two patients with the triplication of the *PRKACA* gene presented at a very young age in comparison with the other patients. However, it remains to be confirmed whether a genomic (tissue-specific) dosage-dependent phenotype indeed exists as another patient (patient 8), who presented at the age of four months with bilateral adrenal hyperplasia, severe early-onset Cushing’s syndrome, and distinct acral soft tissue overgrowth due to cutaneous mucinosis, was identified with a mosaic *PRKACA* duplication. In this boy, the same *PRKACA* duplication was identified in DNA extracted from an affected skin biopsy and resected adrenal tissue but could not be confirmed in blood. The duplication is presumed to have arisen *de novo* as a postzygotic event during early embryogenesis. However, as the variant allele frequency for the duplication in the affected tissues was not reported, the extent of mosaicism could not be further characterized.

The exact pathophysiological mechanisms underlying adrenal tumourigenesis in patients with a *PRKACA* duplication remain largely unknown. Recent Hi-C chromatin conformation capture analyses in patient tumours provided critical insight in the chromatine architecture underlying PPNAD [13]. The analysis revealed that, in addition to PRKACA overexpression, constitutional *PRKACA* duplications can reorganize chromatin topology in adrenal tumours through the formation of newly established topologically associating domains (neo-TADs).

In the majority of the patients with a *PRKACA* duplication, the clinical phenotype seems to be restricted to isolated PPNAD. In some patients extra-adrenal manifestations have been observed. In one female patient (patient 9), the authors indicated a clinical diagnosis of Carney complex, based on the observation of PPNAD and the occurrence of lentigines and small cystic nodules in the thyroid. Given its distinct genetic etiology, this phenotype may be more appropriately characterized as PPNAD mimicking Carney complex rather than Carney complex. In the patient with a mosaic *PRKACA* duplication, the authors suggested that the soft tissue overgrowth of the palms and soles with cutaneous mucinous deposition could be a new distinct entity of *PRKACA*-mediated condition. Noteworthy, the high cortisol levels also likely contributed to the appearance of these skin lesions as these lesions improved after the adrenalectomy. Malignant tumours have been observed in two individuals. Patient 4 was diagnosed with a well-differentiated tubular breast carcinoma and extensive ductal carcinoma in situ at the age of 49 years. The immunohistochemistry was positive for the PRKACA subunit. The index patient in the current study was diagnosed with papillary thyroid carcinoma at the age of 29 years. The immunoexpression of PRKACA and PRKAR1A in the tissue of the thyroid cancer was positive, though weak, which is as expected in non-adrenal organs (data not shown, personal observation by FV). Future studies should indicate whether PRKACA copy-number gain predisposes to thyroid cancer. In addition, given the young age of the patients reported so far, it remains to be defined whether *PRKACA* duplications increase the risk of certain cancers.

In conclusion, PRKACA overexpression due to a germline duplication (or triplication) can underly benign adrenal tumours leading to Cushing syndrome. Improved understanding of the pathophysiological mechanisms of these PRKACA defects will pave the way for the development of personalized molecular therapies targeting the cAMP/PKA signalling pathway for treating cortisol-producing tumours in humans.

## Data Availability

All data supporting the findings of this study are available within the paper .

## Abbreviations

PKA: Protein kinase A
PPNAD: Primary pigmented nodular adrenocortical disease
BAH: Bilateral adrenal hyperplasia
BNAD: Bilateral nodular adrenal disease
ACTH: adrenocorticotropic hormone
T1DM: type 1 diabetes
CNV: Copy Number Variant
ChAS: Chromosome Analysis Suite
WES: Whole Exome Sequencing
